# Protocol: high throughput silica-based purification of RNA from Arabidopsis seedlings in a 96-well format

**DOI:** 10.1186/1746-4811-7-40

**Published:** 2011-12-02

**Authors:** Eliane Salvo-Chirnside, Steven Kane, Lorraine E Kerr

**Affiliations:** 1 The Centre for Systems Biology at Edinburgh (CSBE), the University of Edinburgh, CH Waddington Building, King's Buildings, Mayfield Road, Edinburgh EH9 3JD, UK

## Abstract

The increasing popularity of systems-based approaches to plant research has resulted in a demand for high throughput (HTP) methods to be developed. RNA extraction from multiple samples in an experiment is a significant bottleneck in performing systems-level genomic studies. Therefore we have established a high throughput method of RNA extraction from *Arabidopsis thaliana *to facilitate gene expression studies in this widely used plant model. We present optimised manual and automated protocols for the extraction of total RNA from 9-day-old Arabidopsis seedlings in a 96 well plate format using silica membrane-based methodology. Consistent and reproducible yields of high quality RNA are isolated averaging 8.9 μg total RNA per sample (~20 mg plant tissue). The purified RNA is suitable for subsequent qPCR analysis of the expression of over 500 genes in triplicate from each sample. Using the automated procedure, 192 samples (2 × 96 well plates) can easily be fully processed (samples homogenised, RNA purified and quantified) in less than half a day. Additionally we demonstrate that plant samples can be stored in RNA*later *at -20°C (but not 4°C) for 10 months prior to extraction with no significant effect on RNA yield or quality. Additionally, disrupted samples can be stored in the lysis buffer at -20°C for at least 6 months prior to completion of the extraction procedure providing a flexible sampling and storage scheme to facilitate complex time series experiments.

## Introduction

Systems biology involves the study of molecules in context as part of a larger system or network rather than in isolation and the development of mathematical models of the particular system being studied. Both the wet (laboratory-based) and dry (computer-based) experiments are used to inform each other and together generate a greater understanding of the biological system [1,2]. Over the last few years there has been a movement towards a systems approach to studying biology with a concomitant year-on-year increase in publications to over 1,500 in 2009 [3] and this approach is being used in diverse fields such as stem cell differentiation (reviewed in [4]) and circadian rhythms in plants and animals (reviewed in [5]). As such systems-based approaches become increasingly more popular and incorporated in to a large variety of laboratories, there is a requirement for high throughput (HTP) experimental methods to be developed. As HTP procedures can be arduous for researchers to perform, automation is even more desirable especially when processing very large numbers of samples for an experiment.

Historically, RNA extraction has been performed using organic solvents and phenol-chloroform [6]. Similar liquid-liquid extraction methods using commercially available reagents such as TRIZOL (Invitrogen/Life Technologies) or specifically targeted to more challenging high-polysaccharide-containing samples (such as plants and some Gram-negative bacteria) using CTAB (cetyltrimethylammonium bromide) have been developed [6]. These methods have been widely used, are relatively cheap and simple to perform and isolate large yields of high quality RNA. However, recently there have been concerns expressed over the use of TRIZOL with plant tissue [7,8]. An alternative is to use methods based on solid phase nucleic acid extraction (such as the selective binding of nucleic acid to silica matrices). These methods are commercially available as spin/vacuum-column kits and are convenient and efficient to use [6]. In addition, such silica membrane-based methods eliminate the health and safety issues associated with the use of phenol-chloroform particularly prevalent when processing of large numbers of samples. The recent availability of these kits in a 96 well plate format, which is desirable for ease of handling of multiple samples, also opens up the possibility of automating the procedure using a liquid handling robot. Therefore we have optimised and validated a silica membrane-based HTP method for purification of high quality total plant RNA in a 96 well format from 9-day-old *Arabidopsis thaliana *seedlings. Depending on the available equipment, the method can be performed manually using multichannel pipettes or automated with a liquid handling robot. The developed protocols enable the study of gene expression by qPCR (real time-quantitative PCR) in large numbers of samples (eg time series, different genotypes, treatments etc).

## Materials and methods

### Consumables and Reagents

#### Sample collection

• RNA*later *solution (e.g. Sigma-Aldrich, http://www.sigmaaldrich.com, Cat.# R0901).

• 1.5 ml eppendorf tubes.

#### Sample Disruption

• 2 ml eppendorf tubes (Molecular BioProducts, http://www.mbpinc.com/, Cat.# 3453). NOTE: *If using alternative tubes, ensure that they are sufficiently strong to withstand bead beating in a mixer mill*.

• 5 mm stainless steel cone balls (Retsch, http://www.retsch.com/uk, Cat.# 22.455.0003).

• 0.4 M HCl.

• RNase-free water.

#### RNA Isolation

• illustra RNAspin 96 RNA isolation kit (GE Healthcare, http://www.gelifesciences.com/, Cat.# 25-0500-75 supplied through Fisher Scientific http://www.fisher.co.uk/) which in addition to the binding, wash and elution plates, contains buffers RA1, RA2, RA3 & RA4 and DNaseI. The kit requires the addition of 1% β-mercaptoethanol to buffer RA1 and ethanol to buffers RA3 and RA4 prior to use.

• Filter plate (illustra RNAspin 96 Filter Plate, GE Healthcare, Cat.# 25-0500-88 supplied through Fisher Scientific).

• 1.1 ml 96 deepwell plates (StarLab, http://www.starlab.co.uk/, Cat.# E2896-0110).

##### Specific consumables required for automated protocol only

• Disposable tips for robot (conductive with filter, Tecan Group Ltd, http://www.tecan.com/). To process 1× 96 well plate requires 8× 200 μl tips (i.e. 1 column from a 96 well box, Cat.# 30 000629) and 144× 1000 μl tips (i.e. 1.5× 96-boxes, Cat.# 30 000631).

• Disposable 100 ml and 200 ml troughs for buffers on robot deck (Tecan Group Ltd, Cat.# 10 613 048 and 10 760 646 respectively).

### Equipment

• Oven for preparing beads (e.g. Binder BD-23, http://www.binder-world.com).

• Bead-mill (e.g. TissueLyser, Qiagen, http://www.qiagen.com/).

• Benchtop centrifuge at room temperature capable of speed of 11,000× g for centrifuging lysed solutions in 2 ml tubes (e.g. Eppendorf 5810R centrifuge fitted with an F-45-30-11 rotor).

• Plate centrifuge at room temperature capable of speed of 3,220× g (e.g. Eppendorf 5810R centrifuge fitted with an A-4-81 swing-bucket rotor).

• NanoDrop spectrophotometer (ND1000, ThermoScientific, http://www.nanodrop.com).

Agilent 2100 Bioanalyser (Agilent Technologies, http://www.agilent.com/)

#### Specific equipment required for automated protocol only

**Figure 1:**
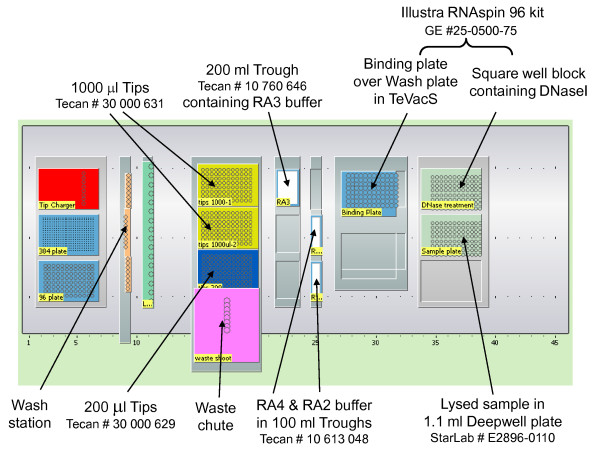
**Schematic diagram of the robot deck layout**. The details of the reagents and plasticware required to carry out the automated RNA extraction procedure are shown with their relative positions on the liquid handling robot deck area.

• Freedom Evo-2 150 liquid handling robot (Tecan Group Ltd, http://www.tecan.com/) with 8-channel liquid handling arm fitted with disposable tips (200 μl and 1 ml as detailed above) and a TeVacS vacuum unit. Although we used the vacuum unit with a custom built spacer to ensure the wash plate (GE Healthcare) was positioned at the correct height directly below the binding plate, a standard size spacer (number 6) can be used. Details of the robot deck layout are depicted in Figure 1. NOTE: *Although our method was developed and performed on a high specification liquid handling robot, it can be easily transferred to other platforms*.

### Growth of *A. thaliana *seedlings

Wildtype *Arabidopsis thaliana *(Ws) seedlings carrying the *ELF3:LUC *transgene (*EARLY FLOWERING 3*) [9] or CCA1-OX (*CIRCADIAN CLOCK ASSOCIATED 1*) seedlings [10] carrying the *CAB2:LUC *transgene (*CHLOROPHYLL A/B-BINDING PROTEIN 2*) [11] were grown on 0.5× Murashige-Skoog (MS) 1.2% agar in 12L:12D under white light (100 μmol·m^-2^·s^-1^) at 22°C for 9 days.

### RNA Quantification

The concentration of purified RNA was measured using a NanoDrop spectrophotometer (ND1000). Typically 2 μl of each sample was used.

### RNA Integrity

The integrity and quality of purified RNA were analysed using an Agilent 2100 Bioanalyzer and a RNA 6000 Nano Assay kit (Agilent Technologies, http://www.agilent.com/) according to the manufacturer's instructions. The ladder used was RNA 6000 ladder (Ambion, http://www.Ambion.com, Cat # AM7152) and 150-200 ng of each sample was loaded. All samples and the ladder were denatured at 70°C for 2 mins prior to analysis. The results were analysed using Agilent 2100 Expert software.

### Reverse Transcription and qPCR analysis

Purified total RNA (1 μg) was reverse transcribed into cDNA using SuperScript VILO cDNA synthesis kit with oligo dT primers (Invitrogen/Life Technologies, http://www.invitrogen.com/) according to the manufacturer's instructions. cDNA was diluted 1/10 and 1 μl used for subsequent qPCR. qPCR analysis for *isopentenyl pyrophosphate:dimethylallyl pyrophosphate isomerase 2 *(*IPP2*) in a final volume of 10 μl was then performed using LightCycler 480 SYBR Green I Master mix on a LightCycler480 (both Roche Applied Science, https://www.roche-applied-science.com/). The following primers each at 300 nM were used: *IPP2 *forward primer GTATGAGTTGCTTCTCCAGCAAAG and *IPP2 *reverse primer GAGGATGGCTGCAACAAGTGT [12,13]. The following qPCR conditions were used: a hot start of 95°C for 5 mins followed by 45 cycles of 95°C for 10 secs, 60°C for 20 secs and 72°C for 20 secs.

## Protocol

**Figure 2:**
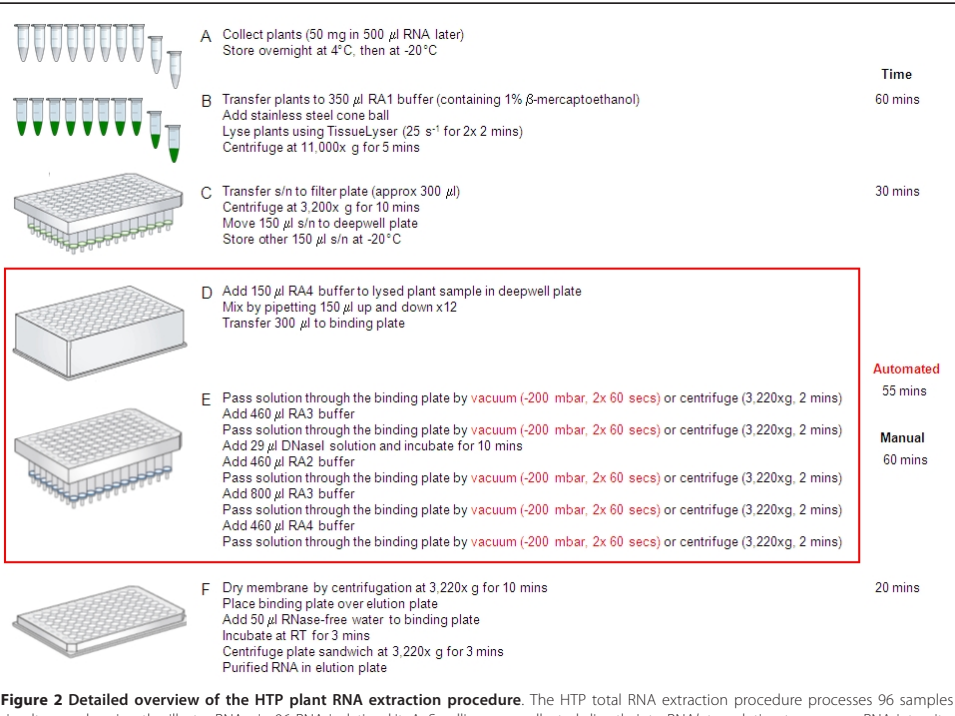
**Detailed overview of the HTP plant RNA extraction procedure**. The HTP total RNA extraction procedure processes 96 samples simultaneously using the illustra RNAspin 96 RNA isolation kit. A: Seedlings are collected directly into RNA*later *solution to preserve RNA integrity. B: Plant tissue is homogenised using a bead mill. C: Lysed samples are filtered to remove debris and half stored in case of catastrophe. RNA is extracted from the other half. D: RNA is prepared for binding to the silica membrane. E: RNA binds to the silica membrane and unbound material is removed by a series of buffer washes. F: Purified RNA is eluted by centrifugation. Sections D and E (boxed) can be performed manually using multichannel pipettes and either centrifugation or vacuum to pass solutions through the binding plate or alternatively automated using a liquid handling robot with a vacuum manifold. The approximate time taken for each step is indicated on the right. Although the automated section is only slightly quicker in terms of actual time taken to complete the procedure, the automated protocol does not require any hands on intervention of the researcher thus freeing up time to complete other work. These factors combine resulting in a 4 fold increase in possible throughput using the automated rather than the manual protocol. Images of the illustra RNAspin 96 kit plates ^© ^2011 General Electric Company - reproduced by permission of the owner.

A detailed method overview is presented in Figure 2.

### Sample collection

The automated RNA extraction procedure is optimal when approximately 20 mg of seedlings are loaded per well of the 96 well purification plate. Loading higher amounts resulted in clogging of the binding membrane and reduced yields. However, 45-50 mg of plant tissue were originally pooled for each sample and partially processed (up to end of Sample Lysis - step 12). Only half of the lysed sample was processed further to complete the RNA extraction procedure while the other half was stored in the freezer (-20°C) after lysis as backup in the case of some experimental catastrophe.

1) Using clean tweezers, harvest 45-50 mg Arabidopsis seedlings per sample directly into 1.5 ml eppendorf tubes containing 500 μl RNA*later*.

2) Store samples overnight at 4°C to allow full penetration of the RNA*later *solution.

3) Store samples at -20°C until sample lysis and completion of the RNA extraction procedure. NOTE*: At this point, samples can safely be stored at -20°C for up to 10 months with no detrimental effect - see comments section below*.

### Sample Lysis

4) Before use, 5 mm stainless steel cone balls (one per sample) are prepared by washing in 0.4 M HCl for 1 h, thoroughly rinsed using RNase-free water and then baked at 240°C for at least 4 h. NOTE: *If the balls are not rinsed properly the acid not only causes rust and corrosion damage to the balls but would also degrade the RNA sample*.

5) Add prepared cone balls to 350 μl RA1 (containing 1% β-mercaptoethanol) in a 2 ml eppendorf tube (one per sample per tube).

6) Carefully remove seedlings from RNA*later *solution and dab dry on some tissue paper.

7) Transfer seedlings to prepared eppendorf tube containing stainless steel cone ball and RA1 lysis buffer (containing 1% β-mercaptoethanol).

8) Homogenise the samples in two batches of 2× 24 tubes using a TissueLyser bead mill at a frequency of 25 s^-1 ^for 2 × 2 mins. The tube positions are rotated between shaking steps as described in the manufacturer's operating manual. NOTE*: Although the TissueLyser equipment we used is capable of processing 192 samples (2 × 96) in 1.5 ml tubes simultaneously, the size of the bead (5 mm stainless steel cone ball) required for efficient sample disruption and homogenisation necessitated the use of 2 ml eppendorf tubes. Consequently, samples have to be processed in two batches of 48. Although it is possible to use smaller stainless steel balls (e.g. 3-4 mm) and thus process samples in larger batches of 96 × 1.5 ml eppendorf tubes, we have found that this only works well with wild type samples or similar. In our hands, smaller bead sizes do not work well with mutant plant samples which often grow poorly and the resulting yield is very low. As our experiments usually include a variety of genetic manipulations, we routinely use 5 mm cone balls for efficient disruption and homogenisation of all samples*.

9) Centrifuge lysed samples at 11,000 × g for 5 mins.

10) Transfer supernatant to a filter plate.

11) Centrifuge filter plate (over a 1.1 ml deepwell plate) at 3,200 × g for 10 mins to remove debris. NOTE*: Missing out this step subsequently results in clogged wells in the binding plate and drastically reduced yields*.

12) 150 μl of the cleared lysed solution is transferred to another 1.1 ml deepwell plate to be processed for RNA extraction while the remainder is stored at -20°C as backup in case of experimental disaster. NOTE: *At this point, lysed samples can be safely stored at -20°C for up to 6 months with no detrimental effects on purified RNA - see comments section below*.

### RNA Isolation (Manual Procedure)

The manual RNA extraction procedure is performed using multichannel pipettes and either a centrifuge or vacuum manifold to pass the solutions through the plate. NOTE: *We have modified the kit manufacturer's protocol to be used on plate centrifuges commonly found in labs (as opposed to the specialised higher speed ones described in the manufacturer's protocol) and all centrifugation steps were carried out in an Eppendorf 5810R centrifuge with an A-4-81 swing-bucket rotor. The plate sandwich formed by the binding plate combined with the plate included in the kit was too deep to be used in our centrifuge set up. Therefore the plate sandwich was composed of a binding plate over a 1.1 ml 96 deepwell plate not the plate provided with the kit. All vacuum steps were performed at -200 mbar for 2 × 60 secs. In our hands, greater pressure resulted in splashing of the sample and possible cross well contamination*.

13) Prepare solutions as detailed in the illustra RNAspin96 kit instructions (making DNaseI solution and adding ethanol to RA3 and RA4 buffer concentrates).

14) Prepare cleared lysed samples (96 × 150 μl, containing half of original plant tissue) for binding to the silica membrane by adding an equal volume of RA4 buffer (150 μl) to each well of 1.1 ml deepwell plate.

15) Mix thoroughly by pipetting up and down 12 times.

16) Transfer samples (300 μl) to the wells of the binding plate.

17) Apply vacuum (-200 mbar, 2 × 60 secs) OR centrifuge (3,220 × g, 2 mins) to pull the solution through the binding plate.

18) Add 460 μl buffer RA3 to each well of the binding plates (to desalt the silica membranes).

19) Apply vacuum (-200 mbar, 2 × 60 secs) OR centrifuge (3,220 × g, 2 mins) to pull the solution through the binding plate.

20) Add 29 μl DNase I solution to the centre of each well of the binding plate and incubate for 10 mins.

21) Add 460 μl RA2 buffer to each well of the binding plate.

22) Apply vacuum (-200 mbar, 2 × 60 secs) OR centrifuge (3,220 × g, 2 mins) to pull the solution through the binding plate.

23) Add 800 μl RA3 buffer to each well of the binding plate.

24) Apply vacuum (-200 mbar, 2 × 60 secs) OR centrifuge (3,220 × g, 2 mins) to pull the solution through the binding plate.

25) Add 460 μl RA4 buffer to each well of the binding plate.

26) Apply vacuum (-200 mbar, 2 × 60 secs) OR centrifuge (3,220 × g, 2 mins) to pull the solution through the binding plate.

27) Proceed to elution (step 30 below).

### RNA Isolation (Automated Procedure)

The automated RNA extraction procedure is performed using a liquid handling robot with an integrated vacuum unit on the robot deck to pass the solutions through the binding plate.

13) Prepare solutions as detailed in the illustra RNAspin96 kit instructions (making DNaseI solution and adding ethanol to RA3 and RA4 buffer concentrates).

14) Prepare the robotic workstation and check that the actual deck layout matches the virtual one shown on the robot script with all labware (plates, buffer troughs, vacuum manifold etc) containing appropriate solutions (RA2, RA3, RA4 buffers, and diluted DNaseI solution) in the correct locations on the robot deck - see Figure 1 for the deck layout for our script.

15) Place 1.1 ml deepwell plate containing 96 lysed samples (150 μl, containing half of original plant tissue) onto the appropriate position of the robot deck.

16) Select appropriate robot script and run which will perform steps 17-28 as detailed below.

17) Prepare lysed samples for binding to the silica membrane by the addition of an equal volume of RA4 buffer (150 μl) and mix thoroughly by pipetting (150 μl) up and down 12 times.

18) Transfer samples (300 μl) to the wells of the binding plate.

19) Apply vacuum (-200 mbar, 2 × 60 secs) to pull the solution through the binding plate.

20) Add 460 μl buffer RA3 to each well of the binding plates (to desalt the silica membranes).

21) Apply vacuum (-200 mbar, 2 × 60 secs) to pull the solution through the binding plate.

22) Add 29 μl DNase I solution to the centre of each well of the binding plate and incubate for 10 mins.

23) Add 460 μl RA2 buffer to each well of the binding plate.

24) Apply vacuum (-200 mbar, 2 × 60 secs) to pull the solution through the binding plate.

25) Add 800 μl RA3 buffer to each well of the binding plate.

26) Apply vacuum (-200 mbar, 2 × 60 secs) to pull the solution through the binding plate.

27) Add 460 μl RA4 buffer to each well of the binding plate.

28) Apply vacuum (-200 mbar, 2 × 60 secs) to pull the solution through the binding plate.

29) Remove binding plate from robot and proceed to elution (step 30 below).

### Elution

Regardless of whether the manual or automated method is used, the same elution procedure is followed.

30) Centrifuge the plate at 3,220 × g for 10 mins to ensure all ethanol is removed prior to elution.

31) Add 50 μl RNase-free water to each well of the binding plate and incubate for 3 mins.

32) Place binding plate over the 96 well elution plate (contained in the illustra kit) and elute purified RNA by centrifugation at 3,220 × g for 3 mins.

## Comments

### Consistency and reproducibility of HTP method

**Figure 3:**
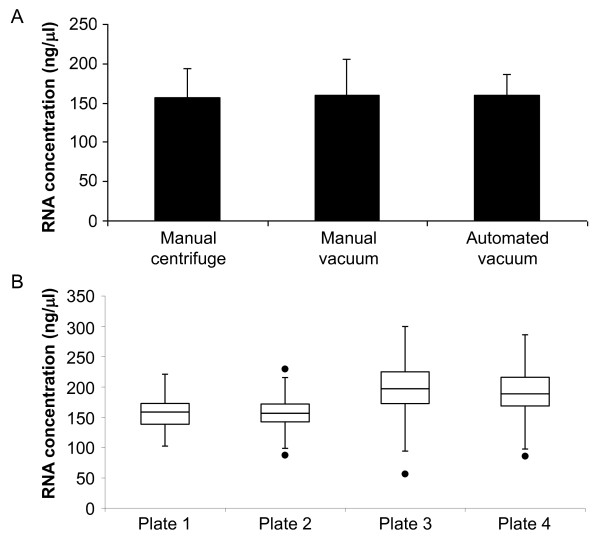
**Consistency and reproducibility of HTP plant RNA extraction procedure**. A: The HTP RNA procedure was performed manually using multi-channel pipettes with either centrifugation or vacuum to pass liquids through the binding plate or automated using a liquid handling robot with a vacuum. All three protocols (manual centrifuge, manual vacuum and automated vacuum) isolated similar amounts of RNA (loading lysate from ~20 mg plant tissue per well). Data are expressed as mean RNA concentration (ng/μl) per well of 96 well plate ± SD. B: Results from 4 different 96 well plates processed using the automated procedure (384 samples) demonstrate that the method is reproducible both intra- and inter-plates. Results are presented as a box and whisker plot of the concentration of RNA isolated (ng/μl; each well containing 50 μl). The bottom and top of the boxes are the 25^th^ and 75^th^ percentiles and the band in the middle is the median. The ends of the whiskers are 1.5 × interquartile range and data points outside this are considered outliers. There are no outliers in plate 1, 1 upper (230 ng/μl) and 2 lower (87 ng/μl and 96 ng/μl) in plate 2, 1 lower outlier (56 ng/μl) in plate 3 and 2 lower outliers (86 ng/μl and 87 ng/μl) in plate 4. The maximum and minimum outliers for each plate (if any) are shown in the box and whisker plot.

The yields of extracted RNA purified using the manual methods (using a centrifuge or vacuum) and the automated (using vacuum) procedure were similar (Figure 3A). Although anecdotal evidence suggests that the yields obtained with centrifugation-based protocols are always greater than those achieved using vacuum manifolds we found no evidence to support this. The reliability and uniformity of the automated procedure was assessed by extracting RNA from 384 plant samples (four 96 well plates). All samples processed on one 96 well plate were prepared from the same experimental batch of 9-day-old seedlings. The automated procedure consistently and reproducibly extracted RNA both within one plate and across plates (Figure 3B). No plate edge effects were evident. The average RNA concentration achieved was 178 ± 42 ng/μl per well over four plates. The average yield was 8.9 μg RNA which was obtained from 20.3 mg of Arabidopsis seedlings comparable with other values reported in the literature using silica-based methods [14]. Although not statistically significant, plants containing different genetic manipulations contained slightly different amounts of total RNA (Figure 3B). The plants processed in plates 1 and 2 were CCA1-OX seedlings containing the *CAB2:LUC *transgene which are elongated [10]. As the longer hypocotyls are due to cell elongation rather than division [15], these 9-day-old seedlings are slightly larger and therefore there were fewer cells per mg of harvested tissue. These samples consequently contain slightly lower amounts of total RNA in comparison to the plants processed in plates 3 and 4 which were wild type (Ws) carrying the *ELF3:LUC *transgene.

### Analysis of Quality of Isolated RNA

**Figure 4:**
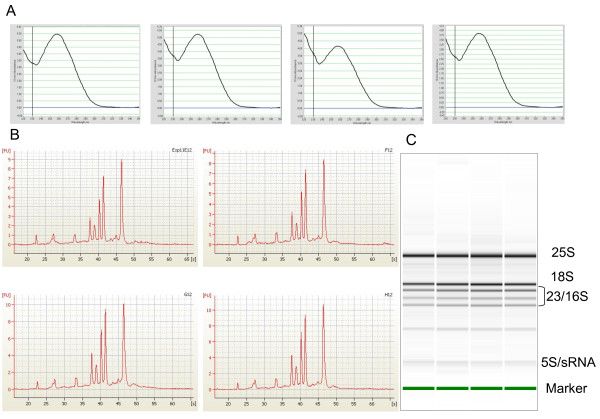
**Assessment of quality of HTP purified RNA**. The quality of the total RNA purified using the HTP RNA extraction procedure was analysed by UV spectrometry using a NanoDrop (A) and an Agilent Bioanalyzer (B, C) which demonstrated the consistent quality of the RNA. A: Measured A_260/280_ ratios for the four samples shown were 2.05, 2.07, 2.03 and 2.02 while the corresponding figures for the A_260/230_ ratios were 1.63, 1.39, 1.12 and 1.44. B: Representative electropherograms and C: corresponding digital electrophoresis gels images show clear sharp ribosomal RNA peaks/bands typical of high quality Arabidopsis RNA. In addition to the cytoplasmic 25S and 18S rRNA peaks, other peaks corresponding to 23S and 16S rRNA from chloroplasts and 5S and small rRNA are evident.

The quality of the HTP purified RNA was assessed by UV spectrometry using a Nanodrop spectrophotometer (Figure 4A). All purified samples had an A_260/280 _ratio of between 1.8 and 2.2 (average 2.09) indicative of highly pure RNA [14]. The A_260/230 _ratios, which are a measure of polysaccharide contamination, were much more variable. This often occurs with plant samples which typically have relatively high polysaccharide contents [16-18] however this would not be expected for 9-day old Arabidopsis seedlings and may reflect other contaminants that have been eluted from the column. Nonetheless, the variable A_260/230 _ratio seemed to have no detrimental effect on subsequent use of the purified RNA for reverse transcription and real time-qPCR (see comments below). This may not be the case for other applications which would have to be individually assessed.

The integrity and quality of purified RNA can be analysed using denaturing gel electrophoresis and visualising the ribosomal RNA bands. However, this technique requires a relatively large amount of RNA to detect clearly visible bands and the RNA is not recoverable for subsequent use. Recently, this analysis has been largely replaced by the use of Bioanalyzers which use microfluidics and capilliary electrophoresis on chips. Bioanalyzers require much smaller amounts of RNA and are ideal when limited amounts of starting sample are available. We therefore assessed the purified RNA using Bioanalyzer microfluidic chips which demonstrated its consistently high quality producing distinct, sharp ribosomal peaks (Figure 4B, C).

### Analysis of Isolated RNA by qPCR

**Figure 5:**
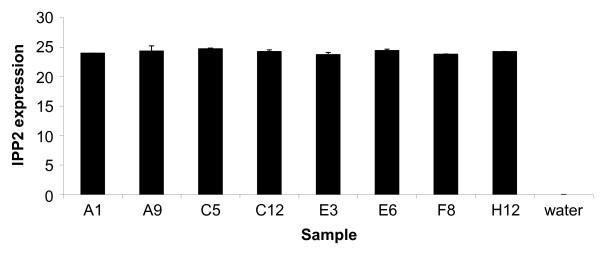
**Suitability of purified RNA for downstream processes**. Total RNA from eight samples purified using the automated HTP RNA extraction procedure was analysed for *IPP2 *expression by reverse transcription and qPCR. No expression was detected in RT- (reverse transcriptase minus) samples (data not shown) or water. Data are presented as the mean ± SD of triplicate qPCR experiments and demonstrate the consistent high quality of the purified RNA.

Plant tissue contains large amounts of polysaccharides and a number of endogenous PCR inhibitors such as polyphenolic compounds which can co-purify with the RNA and inhibit downstream applications such as qPCR [16-18]. The suitability of the purified RNA for such enzyme sensitive methods was therefore assessed by performing reverse transcription followed by qPCR. The minimum usable concentration of RNA permitted in cDNA synthesis reactions using our standard method is 80 ng/μl and this value was easily achieved from all 384 samples on the four plates except one (well A5 in plate 3). However, it was noted during the course of this experiment that this well had a leak. qPCR for *isopentenyl pyrophosphate:dimethylallyl pyrophosphate isomerase 2 *(*IPP2*) gene expression was performed on eight scattered samples (corresponding to well positions A1, A9, C5, C12, E3, E6, F8 and H12) from one of the 96 well plates (Figure 5). The *IPP2 *gene has been identified as an internal housekeeping control for circadian time control studies in Arabidopsis [12,13]. All tested samples were suitable for qPCR analysis and no differences were observed in the levels of expression of *IPP2 *demonstrating the reproducibility and consistency of the automated procedure. The average yield of RNA per well obtained using our HTP procedure (8.9 μg) is sufficient for subsequent cDNA synthesis and analysis of expression by qPCR of over 500 genes in triplicate (based on single-plex qPCR) using our usual procedures [[19] and unpublished results].

### Cost of HTP method

Traditional methods of RNA extraction such as phenol-chloroform are cheaper to perform and produce a greater yield than kit based silica methods (whether in individual spin column format or 96 well format). However, modern health and safety practice encourages the use of safer alternatives (where available) to replace harmful or dangerous chemicals so, even on low throughput, many labs now routinely use silica-based methods (such as spin columns). In addition to simplifying sample handling procedures, the use of a 96 well plate format is cost effective as the cost per sample in the 96 well format is less than half that incurred when using individual mini spin columns. This is before other considerations such as staff time are factored in and represents a considerable saving for larger scale studies involving hundreds of samples. The 96 well plate format also lends itself to automation. Liquid handling robots used to be the preserve of industry however they are becoming more commonplace in academia particularly with the recent move to a more systems-based approach to studying biology. Liquid handling robots are also decreasing in price and becoming more affordable both for core facilities and individual laboratories. Small systems cost around the same as a plate format qPCR machine and can be used for multiple applications as many individual labs will not have sufficient sample throughput for a dedicated robot specifically for RNA extraction. Our optimised protocol can easily be implemented on other robot platforms providing flexibility to the available system.

### Advantages of automation

The automated RNA purification procedure is based on the adsorption of nucleic acids to silica membranes and can be performed on the open bench in contrast to a recently published HTP method using phenol chloroform [8] which requires the use of a chemical fume hood and therefore can not be readily automated. The advantages of using an automated procedure when processing very large numbers of samples are multi-fold. Liquid handling robots perform consistently and continuously resulting in less operator error as even with the most experienced and competent researcher, it is easy to accidentally miss out wells when adding reagents (particularly when performing multiple extractions). In addition, there are no health and safety issues associated with robots performing multiple repetitive tasks and their use reduces the risk of the researcher developing repetitive strain injury (RSI) or work-related upper limb disorders (WRULD). The use of a liquid handling robot frees up skilled experimental hands and time which, in turn, increases productivity. All these benefits also have a positive effect on staff morale as the more routine repetitive liquid handling tasks are removed from the researchers. There is also a considerable time saving element once the automated procedures have been developed and optimised. The time taken to establish an automated procedure is heavily dependent upon the expertise of the researcher but experienced liquid handling robot practitioners could follow our method and write the operation scripts, complete testing and be operational within a few days. Once set up, the automated procedure enables processing, purification and quantification of 192 samples (2 × 96 well plates) in half a day. In contrast, only 96 samples can be fully processed in one day if this method is performed manually using multichannel pipettes. Automation therefore results in a four fold increase in throughput from the manual HTP procedure. Further time savings are possible by increasing the throughput on the post-purification analysis. Although all the work reported here was quantified using a single channel NanoDrop spectrophotometer (which takes approximately one hour for each 96 plate), we have since purchased a system to enable analysis of multiple samples (Infinite 200 PRO NanoQuant, Tecan). The NanoQuant system enables measurement of 16 small volume (2-3 μl) samples at a time and reduces the quantification time for 96 samples to around 20 minutes.

### Storage of Samples after harvesting

**Figure 6:**
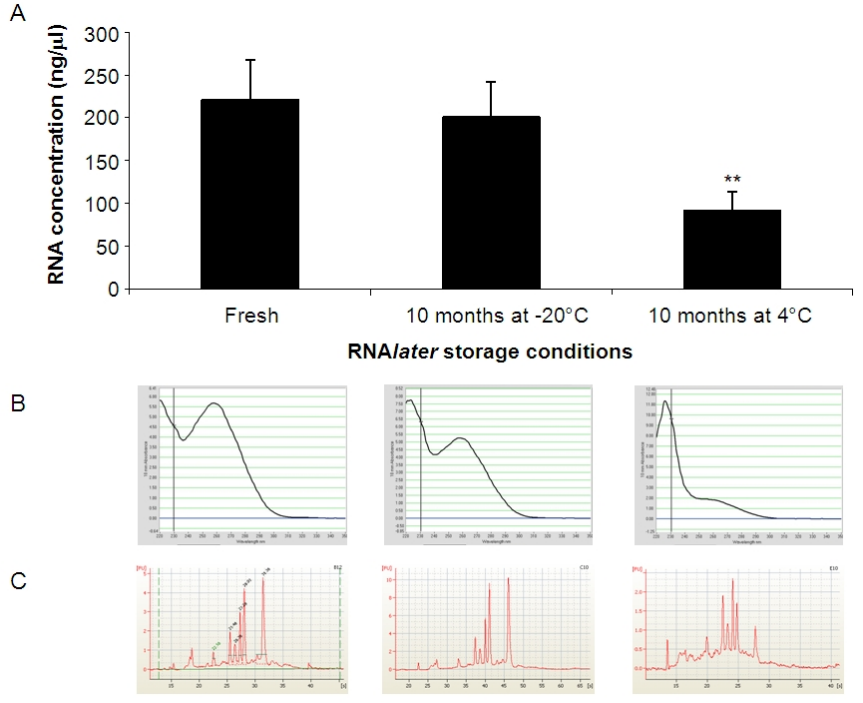
**Seedlings can be stored in RNA*later *for 10 months prior to RNA extraction**. RNA was extracted using the automated HTP procedure from seedlings collected in RNA*later *and processed immediately (minimal storage, n = 96) and from seedlings stored for 10 months at -20°C (n = 48) or 4°C (n = 48). Data are presented as: A: The mean RNA concentration (ng/μl) per well of a 96 well plate ± SD. ** p < 0.01 two-tailed, unequal variance t-test. B: NanoDrop spectrometry scans of three representative samples. C: Electropherograms from bioanalyzer analysis of the same three representative samples.

Many of the issues encountered with plant RNA extraction protocols are thought to originate from the initial sampling procedure [14]. In order to ensure the purified RNA is representative of the tissue being sampled, robust and speedy sampling techniques are required. To ensure integrity of purified RNA, samples either have to be processed immediately after harvest (by lysing in buffer containing stabilising agents and RNase inhibitors) or suspended in time by rapid freezing. The most commonly used method involves snap freezing the sample in liquid nitrogen. This method is relatively simple and can be used as an alternative to the sample collection method we use. However snap freezing poses challenges when using plant samples particularly when processing large numbers of samples over an experimental time course. Samples have to be quickly frozen and remain frozen to prevent degradation of the RNA until the addition of RNA extraction buffers that contain substances to protect RNA from degradation (e.g. lysis buffer RA1 in our protocol which contains guanidine thiocyanate). For plant samples, it is necessary to grind the frozen samples prior to the addition of the lysis buffer or the RNA degrades. Freezing and grinding samples is laborious and time consuming and for large sample numbers, technically challenging to ensure that all samples remain frozen and RNA does not degrade. Alternatively, aqueous solutions for RNA stabilisation (such as the RNA*later *used in our protocol) can be used. These have recently become commercially available and increasingly popular (for example [20]). These solutions rapidly permeate into tissues, stabilising the RNA and preventing its degradation. They have the advantage of being used at room temperature thus removing the requirement to keep the samples frozen to preserve RNA integrity. Therefore their use removes the frozen grinding step in the processing of plant tissue and consequently saves time. In addition, the associated health and safety issues of liquid nitrogen usage are avoided. For all these reasons, we used RNA*later *RNA stabilising solution in our procedure. The manufacturer recommends that samples in RNA*later *are stored at -20°C although storage at 4°C is also possible. We used our automated HTP RNA extraction procedure to independently test these claims for plant tissue and extracted RNA from seedlings stored in RNA*later *for 10 months at -20°C or 4°C (Figure 6). Although RNA can be extracted from seedlings stored in RNA*later *at 4°C for 10 months, the amount recovered is significantly reduced compared to those stored at -20°C for a minimal amount of time (3-4 days) or 10 months (p < 0.01, two-tailed, unequal variance t-test). Storage at 4°C also affects the quality of the RNA.

**Figure 7:**
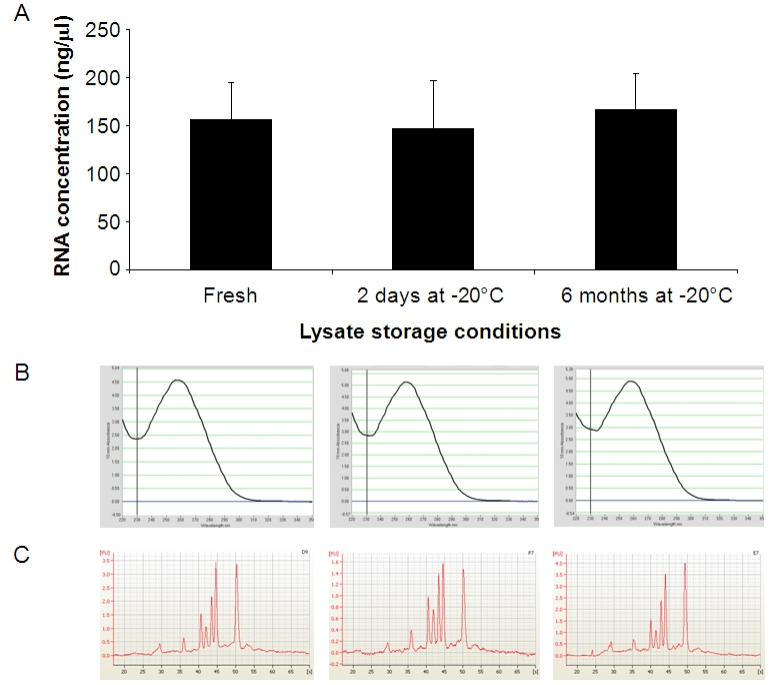
**Lysed samples can be stored for 6 months**. RNA was extracted using the automated HTP procedure from lysed seedlings in RA1 buffer immediately (fresh, n = 96) or after being frozen at -20°C for 2 days (n = 96) or 6 months (n = 96). Data are presented as: A. The mean RNA concentration (ng/μl) per well of a 96 well plate ± SD. B. NanoDrop spectrometry scans of three representative samples. C. Electropherograms from bioanalyzer analysis of the same three representative samples.

We also investigated the effects of freezing the lysed samples in RA1, the lysis buffer supplied with the illustra kit. Samples were processed immediately or after storage at -20°C for 2 days or 6 months prior to completion of the extraction procedure (Figure 7). No significant differences in RNA quantity and quality were observed indicating that samples can be stored as lysates frozen at -20°C for up to 6 months at with no detrimental effects.

## Conclusion

We describe a HTP silica membrane-based method for total RNA extraction from Arabidopsis seedlings in a 96 plate format. This procedure can be performed manually by using appropriate multichannel pipettes or automated with the use of a specialised liquid handling robot. The described protocols isolate RNA in sufficient quantity and of high quality suitable for sensitive downstream processes such as reverse transcription and qPCR. The procedure is time efficient and cost effective for the processing of large numbers of samples. We have used our automated method to investigate and verify sample storage options during the procedure. The combined use of an aqueous RNA stabilising solution (RNA*later*) and the ability to freeze lysed samples with no detrimental affects on the subsequently isolated RNA, provide a flexible sampling and storage scheme. When this is used in conjunction with our automated HTP RNA extraction procedure, it enables complex time series experiments critical for systems-based plant research.

## Competing interests

The authors declare that they have no competing interests.

## Authors' contributions

ESC and SK performed the experimental work and performed the data analysis. LEK conceived and coordinated the project and wrote the manuscript. All authors have read and approved the final manuscript.
